# Effect of listening to music on anxiety, pain, and cardiorespiratory parameters in cardiac surgery: study protocol for a randomized clinical trial

**DOI:** 10.1186/s13063-022-06233-9

**Published:** 2022-04-11

**Authors:** Érica Vieira de Andrade, Vanderlei José Haas, Maíla Fidalgo de Faria, Márcia Marques dos Santos Felix, Maria Beatriz Guimarães Ferreira, Elizabeth Barichello, Patricia da Silva Pires, Maria Helena Barbosa

**Affiliations:** 1 Stricto sensu Graduate Program in Health Care, Federal University of Triângulo Mineiro, Av. Getúlio Guaritá, 107, Uberaba, Minas Gerais CEP: 38025-440 Brazil; 2 Didactic-Scientific Department of Nursing in Hospital Care, Institute of Health Sciences, Federal University of Triângulo Mineiro, Av. Getúlio Guaritá, 107, Uberaba, Minas Gerais CEP: 38025-440 Brazil; 3 Multidisciplinary Health Institute, Federal University of Bahia, Rua Hormindo Barros, 58, Quadra 17, Lote 58, Vitória da Conquista, Bahia CEP: 45.029-094 Brazil

**Keywords:** Anxiety, Postoperative pain, Cardiac surgery, Music, Complementary therapies

## Abstract

**Background:**

Preoperative anxiety and postoperative pain are frequent in cardiac surgeries and constitute important stressors for patients, which can cause several complications. One strategy that aims to alleviate these phenomena is listening to music as a non-pharmacological intervention. The aim of this study is to evaluate the effect of listening to music on preoperative state-anxiety, postoperative pain, at rest and when instructed to cough, and cardiorespiratory parameters in patients undergoing cardiac surgery.

**Methods:**

A randomized, parallel, simple masking clinical trial will be conducted with patients 18 years of age or older who have undergone elective cardiac surgery by sternotomy, who agree to participate in the research and sign a free and informed consent form. Study participants will be randomly divided, in a 1:1 ratio, to one of the two groups: experimental (subjected to listening to music for 20 min in the pre- and postoperative period) or control (standard care in the pre- and postoperative period), using a randomization scheme generated by the Randomization.com website. The sample size calculation was obtained after conducting a pilot study.

**Discussion:**

The results of the study may contribute to the implementation of non-pharmacological interventions in health services, highlighting the protocols for listening to music, to minimize anxiety and pain in cardiac surgery.

**Trial registration:**

ReBEC RBR-8mdyhd. Posted on December 10, 2019

**Supplementary Information:**

The online version contains supplementary material available at 10.1186/s13063-022-06233-9.

## Background

Cardiac surgery is the treatment of choice for many patients with cardiovascular diseases, which are one of the main causes of morbidity and mortality in developed and developing countries. For patients who undergo this surgery, in addition to the pathophysiological aspects, there is also an emotional impact, since the complexity of the procedure and its critical nature can cause anxiety, stress, and depression [1].

Therefore, the psychological component must be inserted in effective preoperative preparation, highlighting the performance and the most active intervention of nursing teams. Preoperative evaluation is essential, starting with contact between nurses and patients, and continuing throughout the surgical process. It must be a complete, holistic assessment, which will result in the formulation of nursing diagnoses and intervention decisions [2].

Anxiety is the most common emotion before cardiac surgeries, and a common nursing diagnosis. Nurses’ preoperative visits are an indispensable procedure, which helps in the psychological preparation of patients and outlines strategies for success of, and fewer complications in, the procedure, both intra and postoperative [3].

Of all types of surgeries, cardiac is the least psychologically accepted by patients, generating high levels of anxiety in the preoperative period, possibly due to the cultural association of the heart as an organ related to life, death and the generation of feelings. The symbolic consideration of the heart and the fear associated with death lead to emotional distress in patients, which can cause complications in the postoperative period [4].

Some studies have evaluated the correlation between preoperative anxiety and postoperative pain in several surgical procedures [5–7]. Several studies [8–13] have shown that pain is very frequent in the postoperative period of cardiac surgeries, which are complex, extensive procedures. It is clear that uncontrolled postoperative pain prevents functional recovery, decreases patients’ quality of life, and becomes a risk factor for persistent postoperative pain, chronic pain, and disability, in addition to showing low quality of care [14].

Currently, the use of multimodal analgesia is recommended to ensure greater efficacy in relieving postoperative pain when compared to single-mode interventions [15]. Among non-pharmacological therapies, which are also covered in multimodal analgesia, music, which has been used in multiple contexts and scenarios of health care, stands out.

Several studies conducted with different objectives have evaluated the effects of musical intervention on the following: pain and anxiety in surgery of patients with breast cancer [16]; stress and anxiety in varicose vein surgery [17]; anxiety and hemodynamic parameters during coronary angioplasty [18]; anxiety in children undergoing elective surgery [19]; perceptions of relaxation, anxiety, and pain in patients during shockwave lithotripsy [20]; and anxiety and pain during dressing changes for burned patients [21], among others.

In the context of cardiac surgeries, research that has analyzed the effects of musical intervention have had conflicting results. A quasi-experimental study in Turkey showed that pain after open heart surgery decreased in patients who listened to music compared to those who did not. Oxygen saturation also increased significantly within the music group, but there was no difference in other physiological parameters [22]. In a survey conducted in Iran, there was also a significant reduction in the intensity of postoperative pain in cardiac surgery among patients who listened to music [23].

However, although other authors [24] have found that music can reduce pain and anxiety in patients with heart disease, in general, the results of studies on the effects of music are inconsistent. A systematic review concluded that listening to music may have a beneficial effect on preoperative anxiety but recommended caution when interpreting results due to the high risk of bias in the included studies [25]. Other researchers also concluded that there is currently insufficient evidence to recommend music after cardiac surgery as an effective non-pharmacological option to reduce pain and anxiety, requiring further studies and standardized intervention protocols [26].

In light of the above, the relevance of this research is that it can serve to minimize existing gaps and add scientific knowledge to the specific area. It is also worth highlighting the importance of evaluating the results of music intervention in postoperative pain, not only at rest, but also when coughing, as this is an important activity in the prevention of complications after cardiac surgery that should be encouraged in the postoperative period. Coughing is one of the activities expected in the postoperative period. It is important because it eliminates the accumulation of pulmonary secretions, thus minimizing the risk of infections and other respiratory complications and reducing the possibility of prolonged surgical recovery. However, the activity of coughing is also considered painful [9, 27].

The hypothesis to be tested in this clinical trial is the following: listening to music in patients undergoing cardiac surgery will promote reduction of the average of anxiety-state scores in the immediate preoperative period; reduction of the average of intensity scores for pain, at rest and when instructed to cough, on the first postoperative day; and reduction in mean systolic and diastolic blood pressure, and heart rate, respiratory rate, and increase in mean oxygen saturation, in the immediate preoperative period and on the first postoperative day.

Therefore, this study has the general objective of evaluating the effect of listening to music on preoperative anxiety-state; postoperative pain, at rest and when instructed to cough; and cardiorespiratory parameters in patients undergoing cardiac surgery.

## Methods

### Objectives

The specific objectives of the clinical trial are:

1. Compare anxiety-state scores between the experimental and control groups in the immediate preoperative period for patients undergoing cardiac surgery;
2. Measure the intensity of acute pain, at rest and when instructed to cough, on the first postoperative day, and identify its location in patients undergoing cardiac surgery;
3. Compare pain intensity scores, at rest and when instructed to cough, between the experimental and control groups, on the first postoperative day of patients undergoing cardiac surgery;
4. Compare cardiorespiratory parameters (blood pressure, heart rate, respiratory rate and oxygen saturation) between the experimental and control groups, in the immediate preoperative period and on the first postoperative day of patients undergoing cardiac surgery;
5. Compare intra-group, preoperative anxiety-state scores, postoperative pain scores, at rest and when instructed to cough, and cardiorespiratory parameters (blood pressure, heart rate, respiratory rate and oxygen saturation) in patients undergoing cardiac surgery;
6. To verify the correlation between preoperative anxiety-state and intensity of postoperative pain in patients undergoing cardiac surgery.

### Study design

The study will be a single-centered, randomized, parallel, superiority clinical trial with simple masking and will follow the Consolidated Standards of Reporting Trials (CONSORT) Declaration—extension for randomized clinical trials of non-pharmacological treatments [28].

This clinical trial protocol was designed in accordance with the Standard Protocol Items: Recommendations for Interventional Trials (SPIRIT) guidelines [29], as evidenced in the checklist (see Additional File 1).

### Study location

The study will be developed at the surgical clinic unit and coronary intensive care unit (ICU) of the Hospital de Clínicas of the Federal University of Triângulo Mineiro (HC-UFTM), located in Uberaba, Minas Gerais, Brazil.

### Inclusion and exclusion criteria for participants

The study will include patients who will undergo elective cardiac surgery, who are 18 years old or older, of both sexes.

Patients with hearing impairment and/or cognitive impairment (recorded in medical records), patients who are using sedative and/or anxiolytic drugs at the time of data collection in the immediate preoperative period, and those who are unable to maintain a dialogue with the researcher to answer the items on the data collection instrument will be excluded from the study.

Patients who met the study criteria and agreed to participate in the research, and were given details about the study, will sign a free and informed consent form.

### Randomization, concealment of allocation, and masking

Study participants will be randomly allocated, in a 1: 1 ratio, to one of the groups, experimental (with music listening) or control (without music listening). The randomization scheme will be generated by using the website Randomization.com (http://randomization.com).

After randomization, a list will be generated, numbered sequentially, for the allocation of participants to the research groups. To ensure concealment of the allocation, the groups to which participants are assigned, according to the generated list, will be inserted in opaque envelopes and numbered sequentially. These envelopes will be sealed and stored in a safe place. They will only be opened by the statistician, at the time of application of music listening or standard care for the corresponding participants. The entire process of randomization and concealment of the allocation will be carried out by the statistician, who will have no clinical involvement in the study.

The clinical trial will be conducted with simple masking, since the researchers responsible for applying the data collection instrument and measuring the variables studied, as well as the surgical and the prescribing teams, will be masked as to the allocation group of each patient. However, due to the nature of the intervention, it will not be possible to mask the research participants, considering that they will be aware of being part of the experimental group, while they are submitted to listening to music.

### Intervention

The non-pharmacological intervention to be evaluated will be listening to music for 20 min, in a 20-min intervention in the immediate preoperative period and another 20-min intervention on the first postoperative day.

The Joanna Briggs Institute, an international research and development organization, synthesized the best available evidence on the use of music as a therapeutic intervention for the management of anxiety or pain related to surgical interventions. They recommend the use of light, non-lyrical music, with 60 to 80 beats per minute, in low tones, preferably strings, with minimal percussion and a volume of 60 dB [30].

The pieces selected for this research, as well as their order, was part of a collection used in a clinical trial on anxiety in blood donors [31], which received technical advice from two music teachers.

The repertoire, which totals 20 min and 28 s, consists of five pieces of classical instrumental music from the Baroque, Classical and Romantic periods, composed between the eighteenth and nineteenth centuries. They are:

- Piece No. 1: Cello Suite No.1, Prelude/Composer: Johann Sebastian Bach (1685-1750)/Instrument: cello
- Piece No. 2: Nocturne. Op. 9 No.2/Composer: Frédéric Chopin (1810-1849)/Instrument: piano
- Piece No. 3: Clarinet Concerto in A Major K 622 Adagio/Composer: Wolfgang Amadeus Mozart (1756-1791)/Instruments: orchestra and clarinet
- Piece No. 4: Gymnopédie No.1/Composer: Erik Satie (1866-1925)/Instrument: piano
- Piece No. 5: The Carnival of the Animals - Le Cygne/Composer: Charles-Camille Saint-Saëns (1835-1921)/Instruments: piano and cello

The sequence of the songs considered a plan to initially have selections with more active, faster elements, matching the level of activity and stress of the listeners, and gradually moving on to slower, less stimulating, more relaxing selections [32].

Classical pieces have common elements of relaxing, calming music, so they are often used in recordings for relaxation [32].

### Experimental group

The patients in the experimental group will undergo listening to music in the immediate preoperative period (up to 24 h before surgery) and on the first postoperative day (24 to 48 h after surgery), totaling two music listening sessions for each patient.

Each music listening session will have a duration of 20 min and will be carried out on headphones with an SD card containing the music recording. The intervention will be conducted by the researcher, who will adjust the volume of the headset, instruct the patients to stay in a comfortable position in the bed, with their eyes closed, and then start listening to the music. The length of the music listening will be timed by the researcher.

### Control group

Patients in the control group will receive standard care in the immediate preoperative period and on the first postoperative day. Standard care will consist of bed rest/sitting in an armchair with routine care, for 20 min, timed by the researcher.

### Criterion for discontinuing the intervention

The intervention with a given participant will only be discontinued upon completion of the study, or their request to withdraw their consent to participate in the study.

### Concurrent care

The study will not interfere with the pharmacological strategy and care of patients, who will receive medications and procedures according to the medical prescription protocol of the study field institution. Information related to painkillers administered to participants will be inserted in the data collection instrument.

### Data collection and management

Data collection will take place at the patient's bedside, once a day, in the immediate preoperative period (up to 24 h before surgery) and on the first postoperative day (24 to 48 h after surgery). The data related to the anesthetic-surgical procedure will be collected from the patient's medical record.

No data will be collected in the immediate postoperative period (first 24 h after surgery), since in this period, patients may still be experiencing residual anesthetic effects, making data collection unfeasible.

#### Data collection instrument

The data collection instrument, developed specifically for this study, was submitted to content validation by five judges with PhDs, and relevant experience in the fields of nursing, teaching and evidence-based research. The instrument will be filled out by a researcher responsible only for data collection, with no participation in the application of the intervention to the participants.

The instrument contains four parts:

- Part I—Identification, sociodemographic and clinical data: initials of the patient's name, medical record number, date of birth, age, sex, ethnicity, location of residence, education, presented comorbidities, previous cardiac surgery, and classification of physical status according to the American Society of Anesthesiologists [33].
- Part II—Refers to the evaluation in the immediate preoperative period: preoperative time; and the following data will be collected before and after the music intervention or standard care: anxiety-state score according to the State-Trait Anxiety Inventory-State (STAI-State), heart rate, systolic and diastolic blood pressure, respiratory rate, and oxygen saturation.
- Part III—Data regarding the anesthetic-surgical procedure, to be obtained from the patient records: surgery performed, anesthetic block, duration of general anesthesia, use of cardiopulmonary bypass, duration of cardiopulmonary bypass, duration of surgery, duration of the anesthetic-surgical procedure, complications in the operating room, and placement of a catheter for postoperative analgesia.
- Part IV—Refers to the evaluation on the first postoperative day. Information on postoperative time, extubation time, presence of chest drains and prescribed analgesic regimens will be obtained. After applying the Ramsay sedation scale, if the score is ≤ 3 points, the following data will be collected before and after the music intervention or standard care:
  - Evaluation of postoperative pain, with the patient at rest and after being instructed to cough: presence of pain, location of pain, pain intensity according to the Numerical Scale, and classification of pain intensity;
  - Values for heart rate, systolic and diastolic blood pressure, respiratory rate, and oxygen saturation.

To assess preoperative state-anxiety, the STAI will be used, prepared by Spielberger, Gorsuch e Lushene [34], translated and validated for Brazilian Portuguese [35]. It consists of two distinct scales that measure two different concepts of anxiety: state of anxiety and trait of anxiety. The STAI anxiety trait scale contains 20 statements that require individuals to describe how they generally feel. The anxiety state scale (STAI-State) also consists of 20 statements, but individuals indicate how they feel at a given moment [36].

For each statement, whose weight varies from one to four points, the individuals must indicate how they feel, indicating one of the four available alternatives; total scores range from 20 to 80 points on each scale [36].

The present research will evaluate only state anxiety, since the objective is to evaluate the level of anxiety in the period before the surgery.

When applying the scale, it should be made clear to the patients that there are no right or wrong answers to the questions and that the answers must correspond to one of the options given [37]. In the case of STAI-State, the answer options are: absolutely not = 1, a little = 2,quite = 3, and very much = 4.

To calculate the total score, a sum must be derived by adding up the scores for the answers given to each question. However, for positive questions, the score must be inverted, that is, if the patient answers 1, a value of 4 is assigned; if they answer 2, a value of 3 is assigned; if they answer 3, a value of 2 is assigned; and if they answer 4, a value of 1 is assigned. On the STAI-State scale, the positive questions that should be scored in reverse are as follows: 1, 2, 5, 8, 10, 11, 15, 16, 19, and 20 [37].

Previous research has found that the scores on the State-Anxiety scale increase in response to various types of tension and decrease as a result of relaxation training [36].

As for cardiorespiratory parameters, a portable pulse oximeter will be used to measure heart rate and oxygen saturation; breathing movements will be observed and counted for 1 min to check the respiratory rate. The measurement of systolic and diastolic blood pressure will be performed by the indirect method of measurement with the auscultatory technique, using a stethoscope and calibrated adult aneroid sphygmomanometer. The blood pressure measurement technique will follow the recommendations of the Brazilian Guidelines on Hypertension [38].

To quantify the intensity of postoperative pain, the Numerical Scale with 11 points will be used, graded from 0 to 10, where 0 means no pain and 10 means the worst pain ever felt. This is one of the validated scales for assessing pain intensity [15]. The Numeric Scale is a one-dimensional instrument that quantifies the severity or intensity of pain, often used because it is quick and easy to apply [39].

Pain intensity will then be classified as: no pain (0), mild pain (1 to 4), moderate pain (5 to 7), and severe pain (8 to 10) [40].

#### Recruitment and data collection procedures

To carry out the data collection, a team will be created, consisting of three nurse researchers linked to the Study and Research Group on Evidence-Based Practice and Patient Safety at UFTM.

One of the researchers will recruit the participants, allocate them to the groups, and conduct the music intervention, or time the standard care time. The other researchers will be responsible for collecting the information in the medical records and for applying the data collection instrument to the patients, before and after the intervention or standard care, in the pre- and postoperative period.

Before the start of data collection, the team will be trained in the entire process and the instruments to be used, to guarantee standardization and the correct execution of the procedures.

For recruitment, every week the researcher will initially obtain from the Cardiovascular Unit of HC-UFTM the identification of the patients who will be submitted to the cardiac surgical procedure, the date of hospitalization, and the date of surgery.

To encourage the recruitment of participants in order to obtain the target sample size, the research will have the support of the institution that is the site of study. Also, the researchers will try to motivate the participants by informing them about the relevance of the research and the need for their involvement in order to help scientific progress occur.

In the immediate preoperative period, in the hospital ward, patients who meet the inclusion criteria will be informed about the research. After receiving detailed information about the study, those who agree to participate will sign two copies of a free and informed consent form, one for the researcher and another for the patient.

After that, the identification, sociodemographic and clinical data of the patients will be collected, followed by the assessment of the state-anxiety level, by applying the STAI-State, and the measurement of the cardiorespiratory parameters (heart rate, systolic and diastolic blood pressure, respiratory rate, and oxygen saturation).

Then, the intervention will be applied (listening to music for 20 min) for patients in the experimental group, or standard care (routine care) for patients in the control group.

After the intervention or standard care, the instruments for assessing the state-anxiety level and cardiorespiratory parameters will be applied again.

On the first postoperative day, in the Coronary ICU, data related to the anesthetic-surgical procedure and information on postoperative time, extubation time, presence of chest drains, and analgesic regimen prescribed on the day will initially be collected.

Then, the Ramsay sedation scale will be applied, and if the score presented by the patient is ≤ 3 points, the instruments for assessing pain (at rest and when instructed to cough) and cardiorespiratory parameters will be applied.

For pain assessment, first with the patients at rest, they will be asked about the presence of pain. If the answer is positive, they will be asked to indicate the location and assign a score for the intensity of their pain, from 0 to 10, according to the Numerical Scale, where 0 means no pain and 10 the worst pain ever felt. So, the pain will be classified as mild, moderate, or severe.

The patients will then be asked to rest their hands on their abdomens, inhale deeply, and cough. They will be asked about the presence of pain when coughing, and if the answer is positive, they will be asked to indicate the location and assign a score for the intensity of their pain, from 0 to 10, according to the Numerical Scale, where 0 means absence of pain and 10 the worst pain ever felt. So, the pain will be classified as mild, moderate, or severe.

After measuring the cardiorespiratory parameters, the intervention will be applied (listening to music for 20 min) for patients in the experimental group, or standard care will be provided for patients in the control group.

After the intervention or standard care, the instruments for pain assessment (at rest and when instructed to cough) will be applied again, according to the procedure described and the cardiorespiratory parameters.

Once a week, the research coordinator will check the consent forms and check the quality of the data collected by the instruments. After meeting with the coordinator, two researchers from the team will enter the data in an Excel® spreadsheet, which will be stored in a password-protected computer, by double entry, to guarantee the validation of the data. Only researchers will have access to the electronic database, collection instruments and consent forms.

**Fig. 1:**
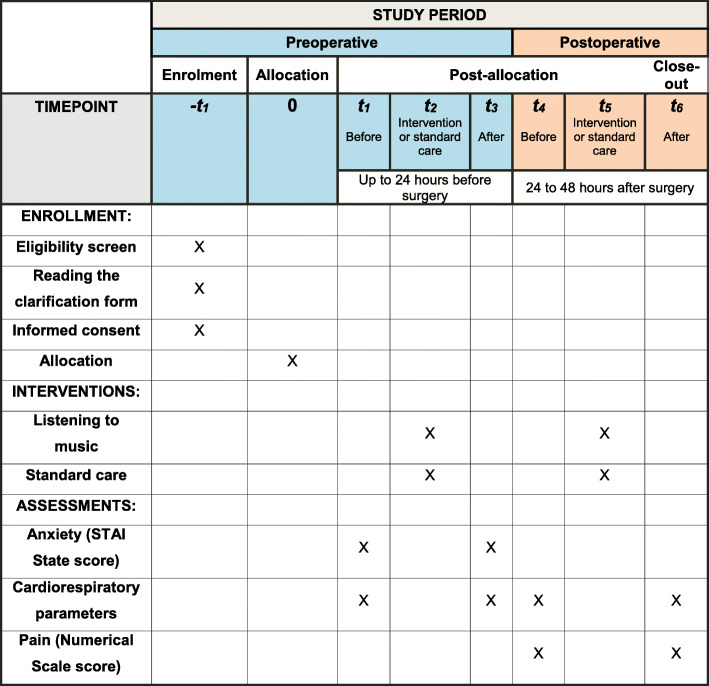
Standard Protocol Items: Recommendations for Interventional Trials (SPIRIT) flowchart

**Fig. 2:**
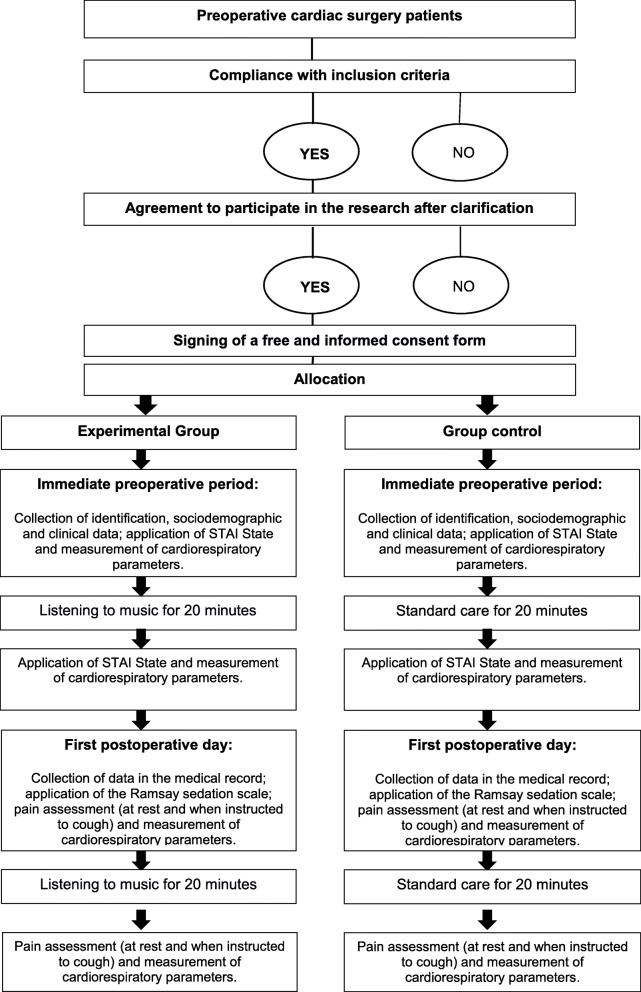
Diagram of the steps of data collection

The flowchart for the research participants is shown in Fig. 1, and the steps of data collection are summarized in Fig. 2.

### Safety protocols

To prevent cross-contamination, the team responsible for data collection will perform hand hygiene before and after the procedures; all equipment that comes into contact with the patients will be disinfected with 70% ethyl alcohol [41]; and after each music listening session, the disposable headphone protectors will be replaced.

During the pandemic period for the new coronavirus, all biosafety protocols will be strictly followed, and the data collection team will use all appropriate personal protective equipment, such as N95 masks, face shields, disposable aprons, gloves, and caps. Patients will also wear disposable masks during collection in the pre- and postoperative period.

As for adverse events, their occurrence is not predictable in this research, given the nature of the intervention and the lack of description in the literature of adverse events resulting from listening to music. However, in the event of any complications or adverse events for the participants, the information will be recorded and the Research Ethics Committee will immediately be notified.

### Evaluated outcomes

#### Primary outcome

- Preoperative anxiety-state: Refers to the average reduction (difference) in anxiety-state scores, obtained after subtracting the scores, before and after intervention or standard care, in both groups (experimental and control), in the immediate preoperative period.

#### Secondary outcomes

- Systolic blood pressure: Refers to the mean reduction (difference) between the values of systolic blood pressure, obtained after subtracting the values, before and after intervention or standard care, in both groups (experimental and control), in the immediate preoperative period and on the first postoperative day;
- Diastolic blood pressure: Refers to the mean reduction (difference) between the values of diastolic blood pressure, obtained after subtracting the values, before and after intervention or standard care, in both groups (experimental and control), in the immediate preoperative period and on the first postoperative day.
- Heart rate: Refers to the average reduction (difference) between the heart rate values, obtained after subtracting the values, before and after intervention or standard care, in both groups (experimental and control), in the immediate preoperative period and on the first postoperative day;
- Respiratory frequency: Refers to the average of the reduction (difference) between the respiratory frequency values, obtained after subtracting the values, before and after intervention or standard care, in both groups (experimental and control), in the immediate preoperative period and on the first postoperative day;
- Oxygen saturation: Refers to the average increase (difference) between the oxygen saturation values, obtained after subtracting the values, before and after intervention or standard care, in both groups (experimental and control), in the immediate preoperative period and on the first postoperative day;
- Postoperative pain: Refers to the mean reduction (difference) of pain scores at rest and when instructed to cough, obtained after subtracting the scores, before and after intervention or standard care, in both groups (experimental and control), on the first postoperative day.

### Statistical methods

#### Sample size

The sample size calculation was performed by a statistician without clinical involvement in the research and obtained after conducting the pilot study, using the Power Analysis and Sample Size (PASS) version 13.0 software. It should be noted that the participants in the pilot study will not be part of the main study. A type I error *α* = 0.05 and a type II error *β* = 0.2 were considered, reaching a statistical power of 80%.

For the pilot study, 13 patients were analyzed, 7 patients from the control group and 6 from the experimental group. The specific objective no. 1 was used to determine the sample size and, considering preoperative anxiety-state as the primary outcome, the mean reduction in the STAI-State scores for the experimental group was 5.67 points (standard deviation: 5.89), and for the control group, it was 3.00 points (standard deviation: 7.30). Based on these values, the sample size calculated is 196 participants, that is, 98 per group.

The estimated dropout rate (loss to follow-up) is 27.8%, considering the data obtained in the pilot study. The sampling loss will be included in a randomization list, and if there is attrition in either of the two evaluated groups, new participants will be included based on this list, until the loss is exhausted. It should be noted that if additional participants are to be randomized to maintain the sample size, each new participant will be randomly assigned to one of the groups with the same original allocation ratio (1:1).

#### Statistical analysis

The collected data will be entered in a Windows® Excel® spreadsheet, using the double entry technique, for later validation. The identification of each study participant, as well as their allocation group, will be done using codes. Then, the validated database will be imported into the Statistical Package for the Social Sciences (SPSS) version 21.0 for processing and analysis.

The normality of the data will be verified by the Kolmogorov-Smirnov test or by the Shapiro-Wilk test. To test the hypothesis of homogeneity of the two groups (experimental and control), when applicable, Student’s *t*-test will be used for independent samples in quantitative variables and the chi-square test of homogeneity for categorical variables.

The univariate exploratory analysis of the data will be performed by the distribution of absolute and relative frequency for categorical variables and measures of central tendency (mean and median) and of variability (amplitudes and standard deviation) for quantitative variables.

To compare the anxiety-state scores, cardiorespiratory parameters, and pain intensity scores, at rest and when instructed to cough, between the experimental and control groups, Student’s *t*-test for independent samples will be used, when applicable.

To compare the anxiety-state scores, cardiorespiratory parameters and pain intensity scores, at rest and when instructed to cough, within each group, experimental and control (intra-group analysis), a paired *t*-test will be used, when applicable.

Subgroup analysis will be performed and, if there is a violation of homogeneity of the groups for one or more confounding variables, a corresponding post hoc multivariate analysis will be performed.

To check the correlation between preoperative anxiety-state and intensity of postoperative pain, Pearson's correlation coefficient will be used, if applicable.

This research will consider a level of significance of *α* = 5% and a confidence interval of 95%. The data will be presented in tables and figures.

### Ethical aspects

The study will be conducted in accordance with the ethical principles of the Declaration of Helsinki and Resolution No. 466, of December 12, 2012, of the National Health Council, Brazil, which deals with the regulatory guidelines and standards for research involving human subjects [42].

The research project was registered and authorized by the Teaching and Research Management of HC-UFTM and approved by the HC-UFTM Research Ethics Committee under Opinion No. 3,474,676, CAAE: 16210819.6.0000.8667. This research was registered in the Brazilian Clinical Trials Registry (ReBEC) platform under number RBR-8mdyhd (UTN: U1111-1238-1227).

Participants will be provided with complete information regarding the research, such as objectives, procedures, risks, and benefits, as well as clarification regarding voluntary participation and the possibility of withdrawing from the study at any time without prejudice to treatment. After reading the clarification form, the participants will sign two copies of a free and informed consent form, one for the participant and one for the researcher.

To minimize the risk of loss of data confidentiality, participants will be identified in the survey by codes. At no time will personal data or any information that can identify participants be disclosed.

All documents generated as a result of the research, such as data collection instruments and forms, will be kept in a safe place, with restricted access. After the completion of the clinical trial, they will remain on file for 5 years, and will then be incinerated, under the responsibility of the coordinating researcher.

It should be noted that any necessary and significant modifications to the study protocol will be documented as an amendment to the protocol and should be formally reported to the Research Ethics Committee, the Brazilian Clinical Trials Registry platform, and the journal in which the protocol is published.

### Access to data

The final data set will not be restricted to researchers and will be made available to the editors and reviewers of the journals, if requested.

### Ancillary and post-trial care

This care will not be necessary in the study. In the event of damage or loss to any participant as a result of their participation in the research, due assistance and compensation, determined by the institution's Research Ethics Committee, will be made available.

### Dissemination of results

The results of the study will be disseminated to the academic and professional community, through release of public access to the thesis, publication of scientific articles, and presentation of papers at scientific events.

## Discussion

This is a protocol for a controlled, randomized clinical trial, with simple masking, that aims to evaluate the effect of listening to music on preoperative state-anxiety, postoperative pain, at rest and when instructed to coughing, and cardiorespiratory parameters in patients undergoing cardiac surgery. This research is justified, in view of the growing number of cardiac surgeries, the complexity of these procedures, the various complications that can be caused by preoperative anxiety and postoperative pain after these surgeries, and due to the need to evaluate and measure these phenomena, as well as proposing non-pharmacological interventions for the relief of patients.

The study is feasible, safe, does not require invasive procedures, and does not interfere with the treatment established for the participants. The intervention will be applied pre- and postoperatively through headphones containing a collection of classical music, pre-selected by the researchers, totaling 20 min and 28 s. The research is also relevant, due to the need for new studies to evaluate the effect of listening to music in the context of cardiac surgery, since the results presented in previous research in the area have not been conclusive, requiring more evidence.

One aspect that also deserves to be highlighted is the fact that the clinical trial evaluates the effect of listening to music on pain, not just when the participants are at rest, but also when they are instructed to cough. This allows a more comprehensive analysis of the intervention’s effectiveness. However, the research has some limitations. It does not allow the participants to choose the pieces to listen to, which may interfere with the outcomes. Also, the listening sessions may not be long enough to generate satisfactory results. In addition, the participants will not be masked, so they will be aware of receiving the intervention. Last, the history of the participants regarding treatment for anxiety and pain before hospitalization will not be considered.

It is hoped that the results of this clinical trial will provide evidence that will contribute to supporting the development and implementation of non-pharmacological interventions, specifically listening to music, in health care, to minimize anxiety and pain, which are considered relevant stressors for surgical patients.

## Clinical trial status

Study protocol version 2 of July 29, 2019. Recruitment of participants will start in November 2021, and this stage is expected to be completed in October 2023.

## Supplementary Information

**Additional file 1.** SPIRIT 2013 Checklist: Recommended items to address in a clinical trial protocol and related documents. A completed SPIRIT checklist with page numbers next to each item corresponding to where it is found within the protocol.

## Data Availability

Not applicable.

## Abbreviations

CONSORT: Consolidated Standards of Reporting Trials
HC-UFTM: Hospital de Clínicas, Federal University of Triângulo Mineiro
ICU: Intensive care unit
PASS: Power Analysis and Sample Size
ReBEC: Brazilian Registry of Clinical Trials
SPIRIT: Standard Protocol Items: Recommendations for Interventional Trials
SPSS: Statistical Package for the Social Sciences
STAI: State-Trait Anxiety Inventory
UFTM: Federal University of Triângulo Mineiro
